# Brain type of creatine kinase induces doxorubicin resistance via TGF-β signaling in MDA-MB-231 breast cancer cells

**DOI:** 10.1080/19768354.2022.2107070

**Published:** 2022-08-31

**Authors:** Seogho Son, Seung-ah Yoo, KeeSoo Nam, Sunhwa Oh, Kyung-min Lee, Jae Youn Yi, Incheol Shin

**Affiliations:** a Department of Life Science, Hanyang University, Seoul, Republic of Korea; b Division of Radiation Effects, Korea Institute of Radiation and Medical Sciences, Seoul, Republic of Korea; c Natural Science Institute, Hanyang University, Seoul, Republic of Korea

**Keywords:** Apoptosis, brain type of creatine kinase (CKB), doxorubicin, MDA-MB-231, transforming growth factor-β (TGF-β)

## Abstract

Brain type of creatine kinase (CKB) regulates energy homeostasis by reversibly transferring phosphate groups between phosphocreatine and ATP at sites of high energy demand. Several types of cancer cells exhibit upregulated CKB expression, but the function of CKB in cancer cells remains unclear. In this study, we investigated the function of CKB in breast cancer by overexpressing CKB in MDA-MB-231 cells. The overexpression of CKB did not affect cell growth rate, cell cycle distribution, ATP level or key mediators of aerobic glycolysis and lactate dehydrogenase isoform levels. Meanwhile, CKB overexpression did increase resistance to doxorubicin. TGF-β-induced Smad phosphorylation and Smad-dependent transcriptional activity were significantly up-regulated by CKB expression without changes in inhibitory Smad protein levels. Moreover, treatment with TGF-β considerably enhanced cell viability during doxorubicin treatment and decreased doxorubicin-induced apoptosis in CKB-expressing MDA-MB-231 cells compared to control cells. These results suggest that CKB attenuates doxorubicin-induced apoptosis and potentiates resistance to doxorubicin by enhancing TGF-β signaling in MDA-MB-231 cells.

## Introduction

Creatine kinase (CK) regulates energy use by reversibly transferring phosphate groups between phosphocreatine and ATP at sites of high energy demand. CK comprises two cytosolic isoenzymes (CK-brain type and CK-muscle type) and two mitochondrial isoenzymes (Wyss and Wallimann 1994). Since CK is responsible for energy homeostasis, it participates in many biological and physiological events (Shin et al. 2007; Salin-Cantegrel et al. 2008; Kuiper et al. 2009). The brain type of creatine kinase (CKB) is upregulated in several cancer cells, including breast cancer cells. However, the role of CKB in breast cancer cells has not been thoroughly investigated.

Transforming growth factor-β (TGF-β) regulates cell proliferation, differentiation, migration, and apoptosis (Welch et al. 1990; Massague et al. 2000). TGF-β is an ambilateral regulator that acts as a tumor suppressor (Akhurst and Derynck 2001) and an oncogenic effector (Wakefield and Roberts 2002). In tumor cells, TGF-β increases oncogenic activities because TGF-β receptors are mutationally inactivated (Massague et al. 2000). As a stimulator of malignancy, TGF-β induces epithelial-to-mesenchymal transformation (Oft et al. 1996) and angiogenesis (Pertovaara et al. 1994) and acts as an immunosuppressor (Torre-Amione et al. 1990). TGF-β also protects against chemotherapeutics in breast cancer cells (Bandyopadhyay et al. 2010).

In this study, we examined the effects of CKB in MDA-MB-231 cells using a retroviral vector to transfer CKB complementary DNA into CKB low-MDA-MB-231 cells. MDA-MB-231 CKB cells withstood doxorubicin-induced apoptosis compared to MDA-MB-231 empty vector cells. In addition, we found that CKB enabled cells to resist doxorubicin-induced apoptosis by potentiating TGF-β signaling.

## Materials and methods

### Cell cultures, antibodies, reagents, and plasmids

293 T cells (ATCC CRL-11268) and MDA-MB-231 cells (ATCC HTB-26) were cultured in Dulbecco’s modified Eagle’s medium (Corning, Lowell, MA) with 10% fetal bovine serum (Corning), 1% penicillin/streptomycin (Gibco, Carlsbad, CA), and 1% antimycotic solution (Gibco). The cells were incubated at 37°C with 5% CO_2_ in a humidified atmosphere. Antibodies to Flag, caspase-3, Bcl-xL, p-Smad2 (Ser465/467), and Smad2 were purchased from Cell Signaling Technology (Beverly, MA). Antibodies to CKB, actin, β-tubulin, LDHA, LDHB, and Smad7 were from Santa Cruz Biotechnology. Sno antibody was purchased from Upstate Biotechnology Inc. (Lake Placid, NY). Doxorubicin was purchased from Calbiochem (Darmstadt, Germany). Recombinant human TGF-β1 was purchased from R&D Systems (Minneapolis, MN). TGF-β type I receptor inhibitor (SB431542) was purchased from TOCRIS (Bristol, UK). 3TP-Lux and pCAGA12-Luc were kindly provided by Jae Youn Yi, Ph.D. (Korea Institute of Radiation and Medical Sciences, Seoul, Korea). NF-κB-Luc (Ju et al. 2011) and FHRE-Luc (Bianco et al. 2003) plasmids were previously described.

### Production of cell lines

The retroviral vector encoding CKB (pWZL-Neo-Myr-Flag-CKB) and the empty vector (pWZL-Neo-Myr-Flag-DEST) were purchased from Addgene (Cambridge, MA). 293 T cells were co-transfected with pWZL-Neo-Myr-Flag-DEST or pWZL-Neo-Myr-Flag-CKB along with gag-pol and VSV-G (8:4:3 ratio). After 48 h, media containing viral soup was concentrated, treated with 8 μg/ml polybrene (Sigma-Aldrich, St. Louis, MO), and added to the MDA-MB-231 cells. After 24 h, the media was replaced with fresh media containing 800 μg/ml neomycin (Sigma-Aldrich) to select infected cells.

### Western blot analysis

Cells were lysed in RIPA lysis buffer (50 mM Tris, pH 8.0, 150 mM NaCl, 1% NP-40, 0.5% sodium deoxycholate, 0.1% SDS). Equal amounts of protein were quantified by BCA protein assay (Thermo Scientific, Logan, UT). Samples were separated on SDS-PAGE gels and transferred to nitrocellulose transfer membranes (Whatman, Dassel, Germany). Membranes were blocked in TBST with 5% skim milk and then incubated with primary antibodies overnight at 4°C, followed by incubation with HRP-conjugated secondary antibodies for 3 h at room temperature. The protein bands were visualized with WEST ZOL plus (iNtRON, Seoul, Korea).

### Quantitative real-time PCR

RNA was extracted with TRIzol reagent (MRC, Cincinnati, OH), and subsequent RT–PCR was performed with a ReverTra Ace qPCR RT Kit (TOYOBO, Osaka, Japan). The expression of CKB was analyzed using the following primers CKB: forward 5’-CCT GGT GTG GGT CAA CGA GGA G-3’, reverse 5’-GCC CAG GTG AGG GTT CCA CAT G-3’. GAPDH expression was used as internal control: forward 5’-TCA GTG GTG GAC CTG ACC TGA CC-3’, reverse 5’-TGC TGT AGC CAA ATT CGT TGT CAT ACC-3’.

### Immunocytochemistry

Cells were washed with PBS, fixed with 4% paraformaldehyde (Sigma-Aldrich), and permeabilized with 0.1% Triton X-100 for 10 min. Fixed samples were blocked with 3% skim milk in PBS for 30 min and then incubated with primary antibody diluted in 1% skim milk in PBS for 1 h. After washing with PBS, cells were incubated with anti-mouse IgG-Cy3 (Molecular Probes, Eugene, OR) then 1 μg/ml Hoechst 33342 was used for DNA staining. Immunofluorescence was observed with an Olympus upright fluorescence microscope (BX50F).

### Proliferation assays

Cells were seeded at 2×10^4^ cells per well in 12-well plates, trypsinized, and counted with a hemocytometer in triplicate every 24 h for 4 days.

### ATP assays

Cells were seeded at 8×10^3^ cells in 96-well plates. After 24 h, CellTiter-Glo reagent was added at a 1:1 volume ratio to cell culture medium and incubated at room temperature for 10 min. The luminescence signal was analyzed with a Varioskan multimode plate reader (Thermo Scientific).

### MTT assays

For 3-(4,5-dimethylthiazol-2-yl)−2,5-diphenyltetrazolium bromide (MTT) assays, cells were seeded in 96-well plates at a density of 5×10^3^ cells per well and treated as indicated. Cells were then incubated with 500 μg/ml MTT (Sigma-Aldrich) solution for 3 h at 37°C.

### FACS analysis

Cells were harvested with 0.25% trypsin and washed with PBS. After centrifugation, cells were fixed in 100% ice-cold methanol for 3 h at −20°C. Fixed cells were incubated with 250 μg/ml propidium iodide (Sigma-Aldrich) and 1 mg/ml RNAse (Sigma-Aldrich) for 10 min. Cell cycle analysis was performed with a BD FACS (Becton & Dickinson Biosciences, San Jose, CA).

### Dual luciferase assays

MDA-MB-231 cells were transfected with 1 μg of reporter construct, and 5 ng of pCMV-RL was used as an internal control. Cells transfected with pCAGA12-Luc and 3TP-Lux were serum-starved for 24 h, then treated with 2 ng/ml TGF-β for 24 h. Cells transfected with NF-κB-Luc and FHRE-Luc were treated with 500 nM doxorubicin for 24 h. Dual luciferase assays were performed according to the manufacturer’s instructions (Promega, Madison, WI).

### Immunoprecipitation assays

For immunoprecipitation assays, 1 mg of cell lysates in lysis buffer was precleared with 30 μl of Protein G Sepharose 4 Fast Flow 50% slurry (GE Healthcare, Piscataway, NJ) for 1 h at 4°C. After centrifugation at 13,000 rpm in a microcentrifuge (Eppendorf, Hamburg, Germany), supernatants were incubated with primary antibody and Protein G Sepharose 4 Fast Flow 50% slurry for 3 h at 4°C. Immunoprecipitates were washed three times with PBS buffer. After supernatants were removed completely, beads were resuspended in SDS sample buffer and boiled at 100°C for 10 min. Samples were separated by SDS-PAGE and subjected to Western blot analysis.

### Bioinformatics

The data for the Kaplan-Meier plot of breast cancer patients were obtained from the Kaplan-Meier Plotter database (http://kmplot.com/analysis/) (Gyorffy et al. 2010; Nagy et al. 2021). The data for the gene set enrichment analysis (GSEA) were obtained from METABRIC (Pereira et al. 2016) and processed in cBioPortal (http://www.cbioportal.org/) (Cerami et al. 2012; Gao et al. 2013).

### Statistical analysis

All experiments were performed in triplicate. Data are presented as means ± standard deviations. Comparison of results from experimental groups versus control groups was done using student’s *t*-test. A value of *p* < 0.05 was marked as a significant difference in each figure.

## Figures and Tables

**Figure 1.**
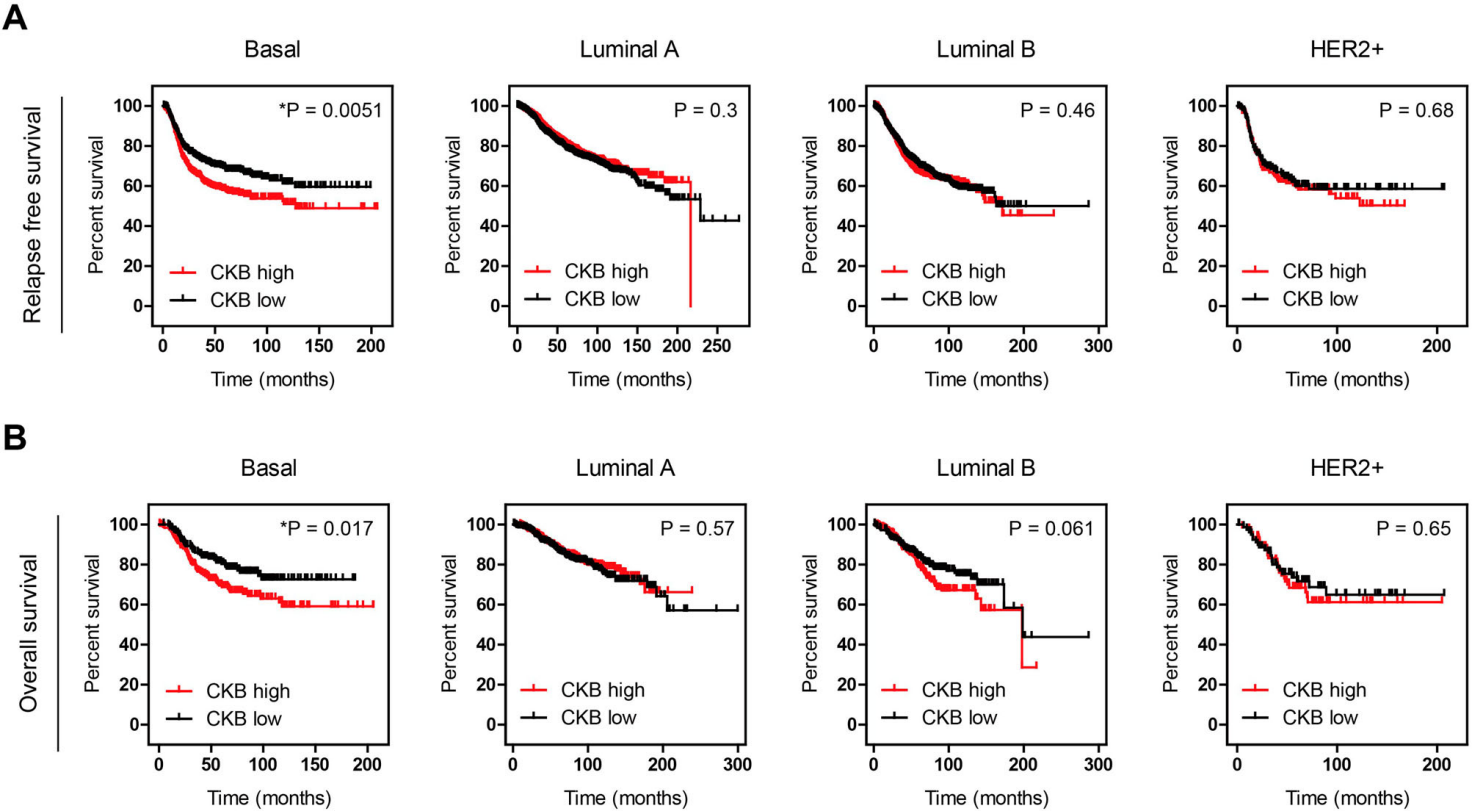
Expression of CKB correlates with poor prognosis in basal-like breast cancer patients. (A and B) Kaplan-Meier plot analysis of each breast cancer subtype using a public database (http://kmplot.com/analysis/).

**Figure 2.**
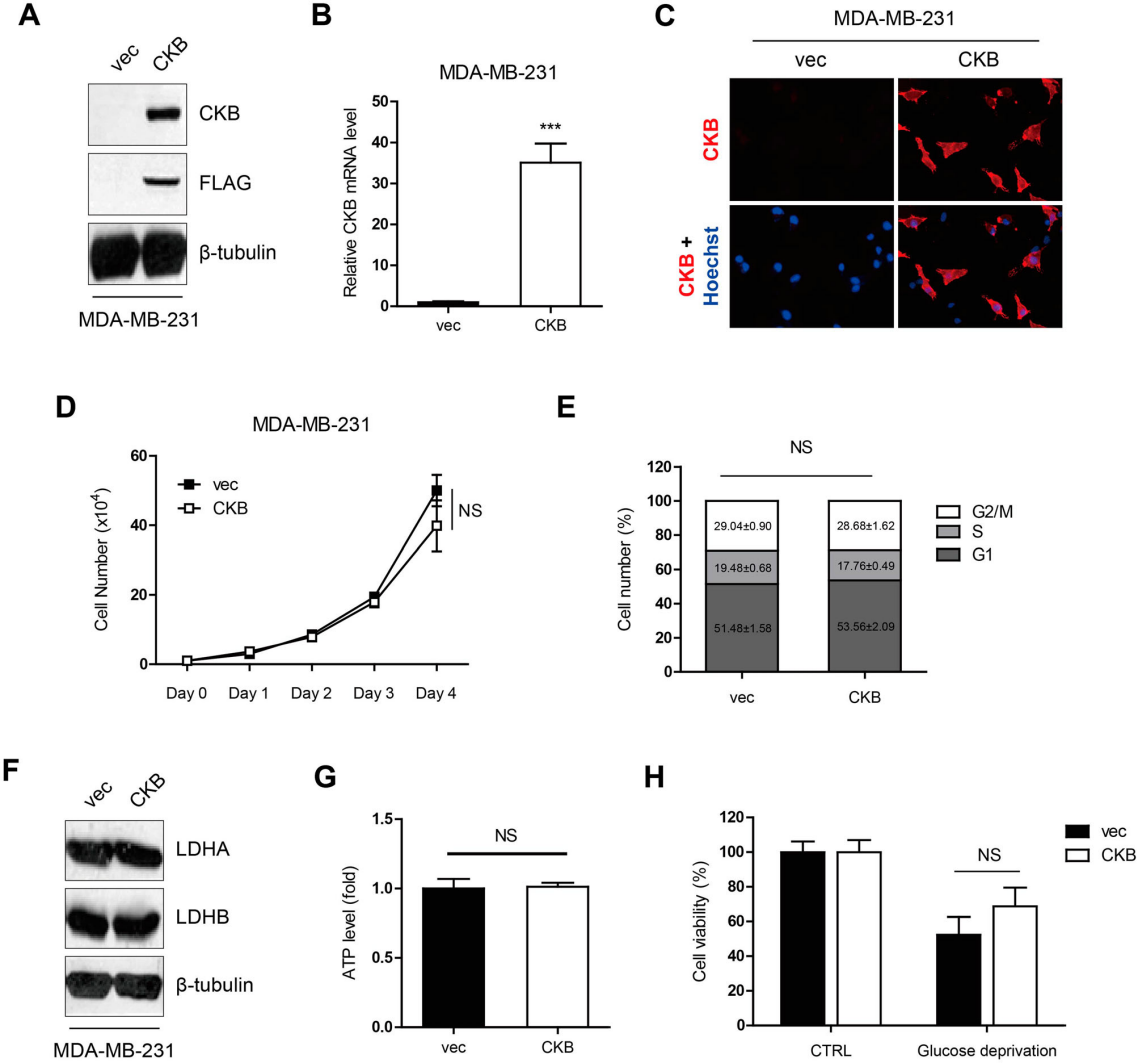
Effects of CKB overexpression on proliferation, cell cycle, LDH signaling, and ATP level. (A) Western blot analysis was used to detect expression of creatine kinase B (CKB) and flag protein. β-tubulin was used as an internal control. (B) Expression level of CKB mRNA was detected by real-time PCR (*P < 0.05). (C) CKB expression was monitored by immunocytochemistry. MDA-MB-231 vec and MDA-MB-231 CKB cells were fixed and stained with CKB antibody and Hoechst dye for DNA. (D) Cells were seeded at a density of 2×10^4^ cells in 12-well plates, and cell proliferation rates were determined by counting for 4 days. (E) Cell cycle analysis was performed using flow cytometry with MDA-MB-231 vec and MDA-MB-231 CKB cells. (F) Western blot analysis was used to detect protein expression of LDHA and LDHB. (G) Cells were seeded at 1×10^4^ cells in a 96-well plate. After 24 h, the cells were subjected to ATP assays. (H) Cells were incubated with 10% FBS/DMEM with or without glucose and subjected to MTT assays.

**Figure 3.**
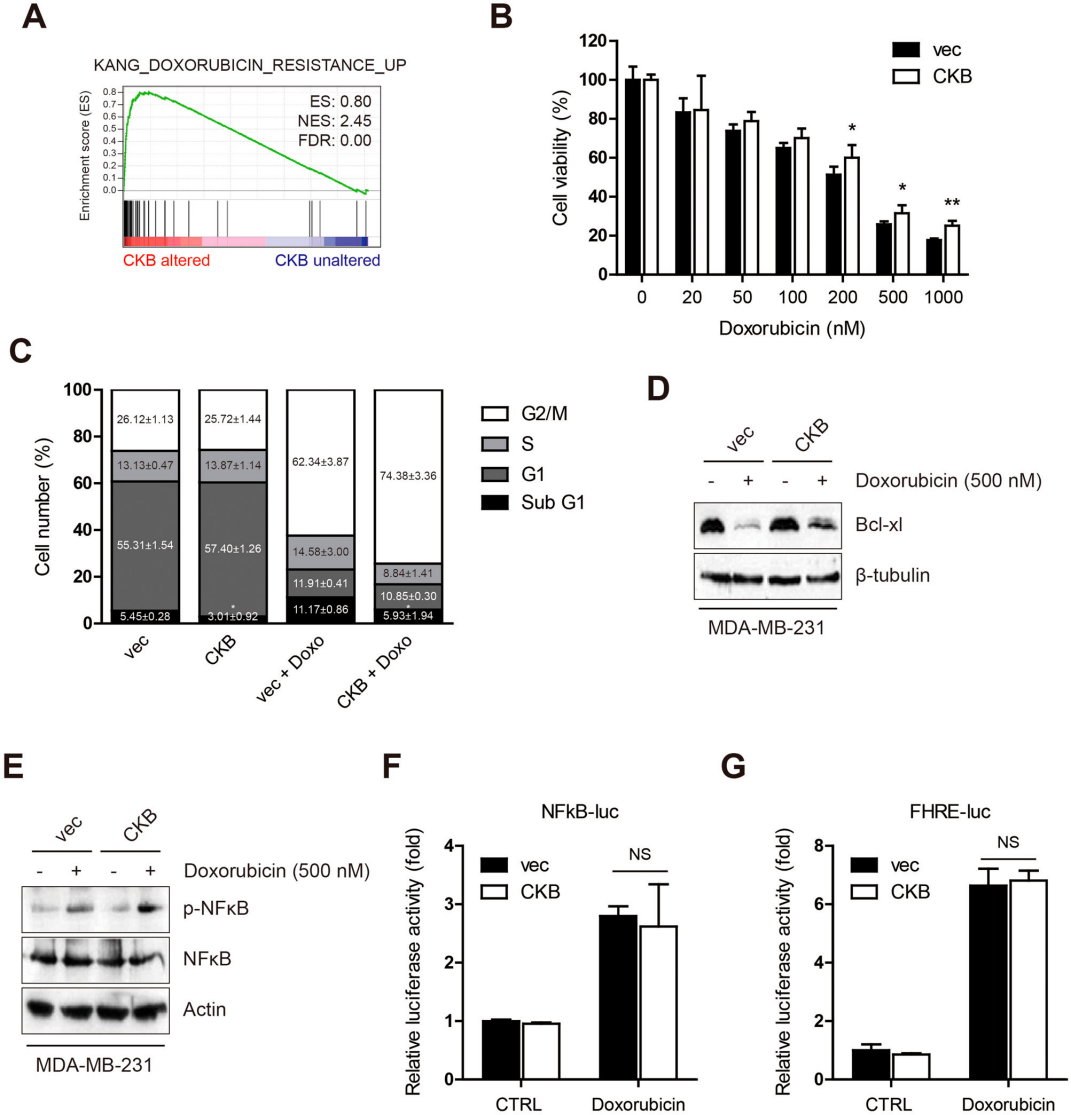
Effects of CKB overexpression on resistance to various stresses in MDA-MB-231 cells. (A) Gene set enrichment analysis (GSEA) performed with the METABRIC dataset. Patients with amplified CKB gene or high mRNA expression were classified as CKB altered, and patients who did not are classified as unaltered (ES: enrichment score; NES: normalized enrichment score; FDR: false discovery rate). (B) MDA-MB-231 vec and MDA-MB-231 CKB cells were treated with various doses of doxorubicin for 48 h and subjected to MTT assays (*P < 0.05) (C) After 24 h exposure to doxorubicin (500 nM), cells were stained with propidium iodide for FACS analysis of cell cycle distribution (*P < 0.05). (D and E) MDA-MB-231 vec and MDA-MB-231 cells were treated with 500 nM doxorubicin for 24 h. Cells were subjected to Western blot analysis with the indicated antibodies. (F and G) Cells were transfected with NF-κB reporter construct or FHRE reporter construct and pCMV-RL as an internal control, then were treated with 500 nM doxorubicin for 24 h, and dual luciferase assays were performed.

**Figure 4.**
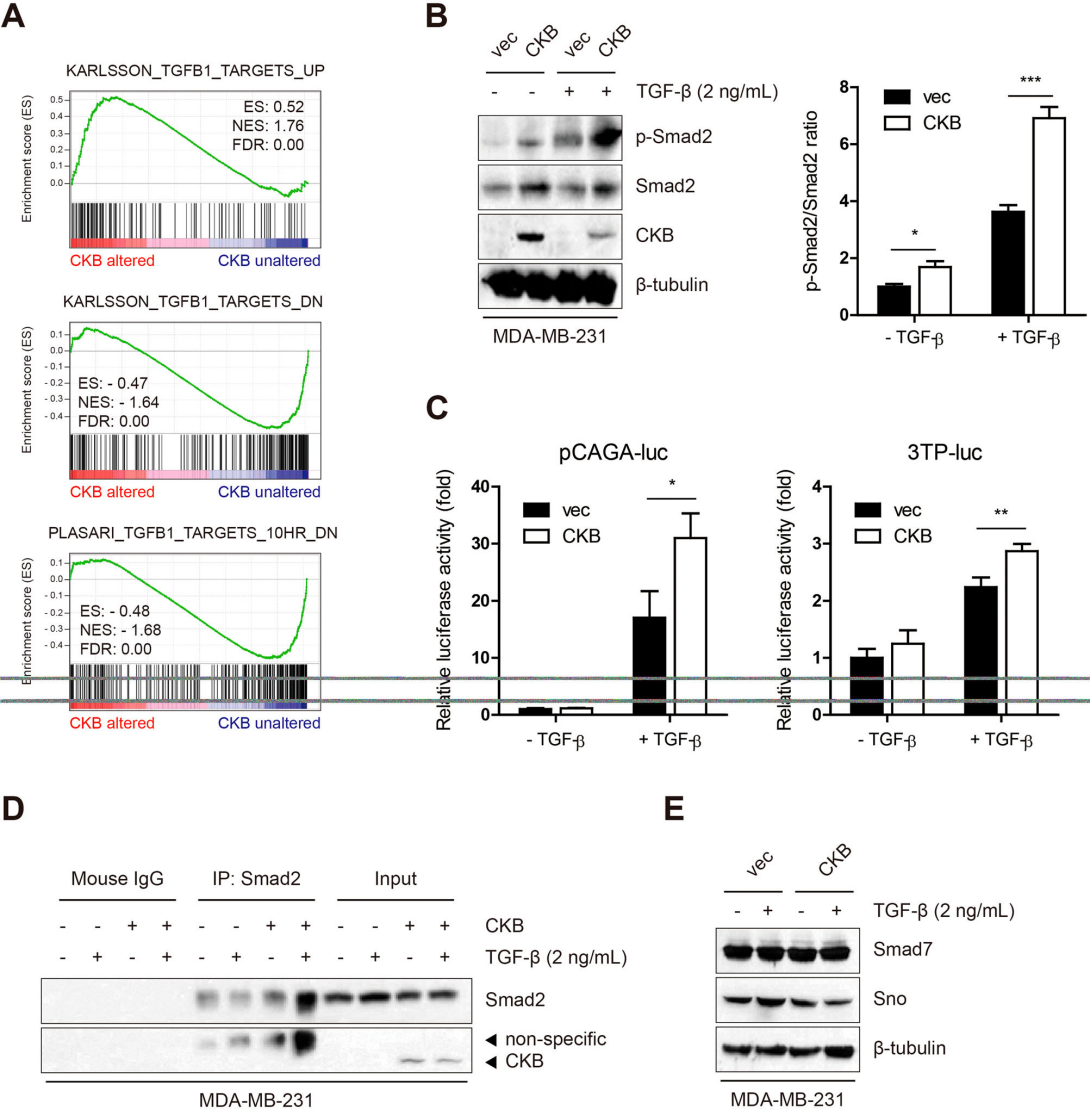
CKB overexpression indirectly potentiates TGF-β signaling. (A) Gene set enrichment analysis (GSEA) performed with the METABRIC dataset. Patients with amplified CKB gene or high mRNA expression were classified as CKB altered, and patients without were classified as unaltered (ES: enrichment score; NES: normalized enrichment score; FDR: false discovery rate). (B and E) After serum starvation for 24 h, MDA-MB-231 vec and MDA-MB-231 CKB cells were treated with 2 ng/ml TGF-β for 24 h. Cells were harvested and subjected to Western blot analysis with the indicated antibodies. The p-Smad2/Smad2 ratio in MDA-MB-231 cells was quantified. (C) Cells were seeded at a density of 1×10^5^ in 12-well plates and transfected with pCAGA12 or 3TP lux reporter construct and pCMV-RL as an internal control. The cells were serum-starved for 24 h, then treated with 2 ng/ml TGF-β for 24 h, and dual luciferase assays were performed (*P < 0.05). (D) Cell lysates were immunoprecipitated with Smad2 antibody and analyzed by Western blotting with Smad2 and CKB antibodies.

**Figure 5.**
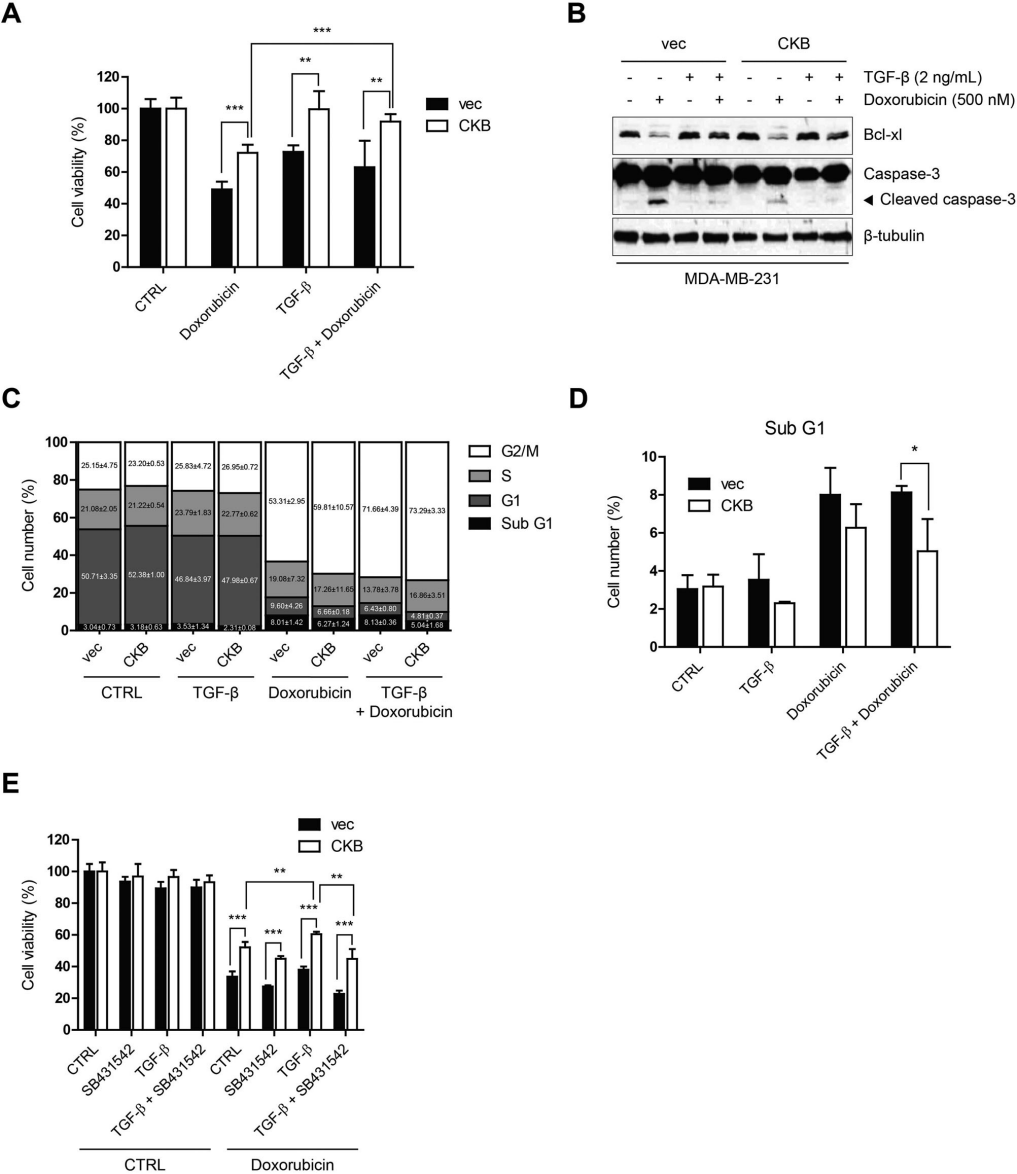
CKB attenuates doxorubicin-induced apoptosis by regulating TGF-β signaling in MDA-MB-231 cells. (A) Cells were treated with 500 nM doxorubicin with or without 2 ng/ml TGF-β and then subjected to MTT assays (*P < 0.05). (B) MDA-MB-231 vec cells and MDA-MB-231 CKB cells were treated with 500 nM doxorubicin with or without 2 ng/ml TGF-β and subjected to Western blot analysis. (C) After 24 h of exposure to 100 nM doxorubicin with or without 1 ng/ml TGF-β, cells were stained with propidium iodide for FACS analysis of cell cycle distribution. (D) The apoptotic cell fraction (sub-G1) was calculated (*P < 0.05). (E) Cells were treated with 500 nM doxorubicin with or without 1 ng/ml TGF-β and 0.5 μM SB431542, then subjected to MTT assays (*P < 0.05).

## Data Availability

All datasets used in this study are publicly available.

## Results

### CKB expression correlates with poor survival of basal-like breast cancer patients

To determine the clinical significance of CKB on breast cancer, Kaplan-Meier plot analysis (KM plot) was performed using a public database. Interestingly, KM plot results showed that CKB expression led to worse prognosis in the basal-like breast cancer patient group (Figure 1A-B), in terms of both relapse-free survival (RFS) and overall survival (OS). Other breast cancer subtypes did not show significant prognostic differences in by CKB status. These findings suggest that high expression of CKB is associated with poor outcome in patients with basal-like breast cancer.

### Effect of CKB overexpression on MDA-MB-231 human breast cancer cells

To determine the function of CKB in basal-like breast cancer cells, CKB was stably overexpressed by retroviral infection in MDA-MB-231 breast cancer cells. MDA-MB-231 CKB-overexpressed cells (CKB cells) exhibited increased CKB expression compared to MDA-MB-231 empty vector cells, which was confirmed at both mRNA and protein level (Figure 2A-C). Since CK is known to be involved in cell cycle regulation, we examined whether CKB affects the proliferation of breast cancer cells (Yan 2016). CKB overexpression did not appear to affect cell proliferation rate (Figure 1D). Similarly, the results of cell cycle distribution analysis using flow cytometry did not reveal any significant difference between the cell lines (Figure 1E). We also determined the effect of CKB on cellular metabolism because CKB is associated with energy homeostasis (Wallimann et al. 1992). Since CKB modulates metabolic pathways, such as glycolysis (Li et al. 2013), which plays a critical role in cancer cells, a key mediator of aerobic glycolysis, lactate dehydrogenase (LDH) (Warburg 1956), isoform levels were assessed. Cancer cells with anaerobic glycolytic phenotype often up-regulate LDHA while down-regulating LDHB (Shim et al. 1997). Nonetheless, the results showed that CKB did not alter the protein levels of LDHA and LDHB (Figure 2F). Although CKB directly regulates ATP level (Wyss and Wallimann 1994), ATP level in CKB cells did not differ from that in vector control cells (Figure 2G). Also, when we examined cell viability under glucose deprivation by MTT assays, the viability of CKB-overexpressing cells did not differ significantly from that of vector control cells upon glucose deprivation (Figure 2H). These data suggest that stable overexpression of CKB does not alter proliferation and cellular metabolism in MDA-MB-231 cells.

### CKB confers resistance to doxorubicin in MDA-MB-231 cells

Next, we conducted gene set enrichment analysis (GSEA) to investigate the role of CKB in breast cancer. Using the METABRIC database, gene expression patterns were compared between patients with amplified CKB gene or high mRNA expression level and those without amplified CKB or CKB overexpression. Interestingly, we found that the gene set related to doxorubicin resistance was expressed at significantly higher level in the CKB-altered patient group than control group (Figure 3A). To investigate whether CKB is related to doxorubicin resistance in breast cancer cells, we compared cell viability between vector control and CKB cells after treatment with doxorubicin. CKB overexpression enhanced the viability of doxorubicin-treated cells compared with vector-control cells (Figure 3B).

To further confirm the inhibitory effect of CKB on doxorubicin-induced apoptosis, we analyzed the sub-G1 apoptotic cell fraction by flow cytometry (Figure 3C). CKB attenuated the increase in the apoptotic cell fraction induced by doxorubicin treatment, while vector control cells had a higher apoptotic cell fraction. Having confirmed that CKB inhibits doxorubicin-induced apoptosis, we further investigated the possible anti-apoptotic signal induced by CKB.

To determine the mechanism of doxorubicin resistance induced by CKB, we measured the expression of apoptotic proteins. When the level of Bcl-xL, an anti-apoptotic protein, was examined, forced expression of CKB increased the Bcl-xL level in doxorubicin treated cells (Figure 3D). NF-κB signaling has been reported to be modulated by doxorubicin (Das and White 1997), and NF-κB regulates Bcl-xL expression (Chen et al. 2000). Therefore, we speculated that NF-κB signaling might be differentially modulated between CKB cells and vector control cells. NF-κB activation was increased by doxorubicin treatment, but the increase in NF-κB activity as determined by phospho- NF-κB level, was not significantly different between CKB cells and vector control cells (Figure 3E). Also, NF-κB-dependent transcriptional activities after doxorubicin treatment were not significantly different between CKB and vec cells (Figure 3F). Therefore, we assumed that Bcl-xL, might be regulated by NF-κB-independent mechanism(s). Next, we checked whether the drug-resistance might be conferred by the Foxo transcription factors which were known to regulate apoptosis-related genes (Kayal et al. 2010). Indeed, FOXO-induced transcription were increased after doxorubicin treatment (Hui et al. 2008) but they were not affected by CKB expression (Figure 3G).

### CKB overexpression potentiates TGF-β signaling in MDA-MB-231 cells

Next, to find the mechanism of doxorubicin resistance by CKB expression, we performed GSEA analysis between CKB altered and CKB unaltered samples. We found that the gene sets related to the TGF-β signaling pathway were highly expressed in patients with amplified CKB (Figure 4A). According to previous studies, TGF-β signaling modulates cell survival (Lei et al. 2007) and apoptosis (Lei et al. 2002). To investigate the functional connection between CKB and TGF-β signaling, we treated vector and CKB cells with human recombinant TGF-β and compared activation of TGF-β downstream signaling between the cell lines. The basal level of phosphorylated Smad2 was significantly up-regulated in cells overexpressing CKB (Figure 4B). After TGF-β treatment, Smad2 phosphorylation increased in both vector and CKB cells, but the increase in Smad2 phosphorylation was substantially higher in CKB cells than in vector cells. To confirm that TGF-β signaling is efficiently up-regulated in CKB cells upon TGF-β treatment, we measured Smad-dependent transcription using 3TP-lux and pCAGA12 luciferase reporter assays (Figure 4C). The results show that overexpression of CKB significantly increases TGF-β induced luciferase activities. To explain the possible mechanism of CKB-induced up-regulation of TGF-β signaling, we determined whether CKB can physically interact with Smad2 to modulate its transcriptional activity. Co-immunoprecipitation assay revealed that but, Smad2 and CKB do not directly associate each other in CKB overexpressing cells (Figure 4D). These data may indicate that CKB modulates TGF-β signaling in indirect mechanism(s).

We also investigated the possibility that CKB may inhibit a negative regulator of TGF-β signaling. When the effect of CKB overexpression on protein level of the inhibitory Smad protein, Smad7 (Yan et al. 2009), and a negative regulator protein, Sno (Deheuninck and Luo 2009), were examined, it was found that the inhibitory molecules of TGF-β signaling were not changed by CKB overexpression (Figure 4E).

### CKB attenuates doxorubicin-induced apoptosis via TGF-β signaling in MDA-MB-231 cells

To check whether CKB-induced up-regulation of TGF-β signaling can rescue MDA-MB-231 cells from doxorubicin-induced apoptosis, MDA-MB-231 vector control and CKB cells were treated with doxorubicin in the presence or absence of TGF-β. We found the enhanced protective effect of TGF-β treatment on doxorubicin-induced cell death by CKB overexpression (Figure 5A). TGF-β indeed enhanced cell viability after doxorubicin treatment in CKB cells compared with vector control cells. We also measured apoptotic proteins after treatment with doxorubicin and TGF-β. Upon treatment with doxorubicin alone, the level of proteolytic cleaved caspase-3 decreased, and Bcl-xL level increased in CKB cells compared with vector control cells (Figure 5B, lanes 2 vs. 6). When cells were co-treated with doxorubicin and TGF-β, the cleaved caspase-3 and Bcl-xL levels were similar to control levels (Figure 5B, lane 4). When cells were pretreated with TGF-β followed by doxorubicin treatment, the sub-G1 apoptotic fraction decreased significantly in CKB cells compared to vector control cells (Figure 5C-D). To ensure that this increased cell survival during doxorubicin treatment was mediated by TGF-β signaling, we used SB431542, a TGF-β type I receptor inhibitor, to block the TGF-β signaling. The viability of CKB cells co-treated with doxorubicin and TGF-β was higher than that of CKB cells treated with doxorubicin alone (Figure 5E) When SB431542 was added to cells co-treated with doxorubicin and TGF-β, cell viability was decreased similar to the level of doxorubicin treatment alone. Altogether, these results indicate that overexpression of CKB enhances TGF-β signaling induced cell survival in MDA-MB-231 cells.

## Discussion

CKB is a brain-type isoform of creatine kinase, which catalyzes the reversible phosphorylation between creatine and ATP (Wallimann et al. 1992). CKB plays a role in energy buffering (Wallimann et al. 1992). In sites of high energy demand, such as the brain, CKB provides cells with ATP by transferring phosphate from phospho-creatine to ADP (Wyss and Kaddurah-Daouk 2000). CKB facilitates cell motility through local ATP generation (Kuiper et al. 2009). Also, CKB strengthens cell adherent junctions producing ATP after HIF-2 dependent expression (Glover et al. 2013). Contrary to our expectations, we did not find differences in cell proliferation, cell cycle distribution, LDH expression, or intracellular ATP level between control and CKB-overexpressing cells (Figure 2).

In contrast to other growth factor families, TGF-β is reported to suppress cell proliferation and regulate apoptosis (Bhowmick et al. 2004). TGF-β signaling is involved in inhibition of apoptosis in MDA-MB-231 breast cancer cells (Lei et al. 2002), and in a recent study, Lu et al showed that TGF-β signaling was significantly changed in pancreatic stellate cells treated with chemotherapeutic agent gemcitabine (Lu et al. 2022). Also, we found that CKB-overexpressing cells showed increased tolerance to doxorubicin-induced cell death. Therefore, we speculated that CKB may potentiate resistance to apoptosis by enhancing TGF-β signaling (Figures 4 and 5). TGF-β signaling is positively regulated by increased ligand- and receptor-related signaling molecules (Miyazono 2000). In some cells, TGF-β receptor expression can be increased by ligand stimulation (Bloom et al. 1996). TGF-β signaling is also positively modulated through other signaling pathways (Miyazono 2000). In the non-Smad pathway, TGF-β activates MAPK signaling including Erk (Hartsough and Mulder 1995), c-Jun N-terminal kinase (JNK) (Hocevar et al. 1999), and p38 MAPK (Adachi-Yamada et al. 1999). Smad-dependent TGF-β signaling is modulated by protein kinase C (PKC) (Yakymovych et al. 2001). Phospholipase Cγ is involved in PKC-dependent Smad phosphorylation and abrogates Smad3 binding to DNA and leads to down-regulation of Smad-dependent TGF-β signaling (Yakymovych et al. 2001). We found that overexpression of CKB increased Smad2 phosphorylation (Figure 4). Therefore, we speculated that CKB potentiates TGF-β signaling in a Smad-dependent manner. Since CKB uses the various phosphogens as substrates (Bessman and Carpenter 1985), we could not completely rule out the possibility that CKB directly phosphorylates Smad2.

In conclusion, we found that CKB prevented MDA-MB-231 cells from undergoing doxorubicin-induced apoptosis. Furthermore, CKB attenuated doxorubicin-induced apoptosis through a TGF-β-dependent mechanism. Although we speculate that CKB regulates TGF-β signaling in a Smad2-dependent manner, the molecular connection between CKB and TGF-β signaling has not been elucidated. Further investigations are needed to explain the link between CKB and TGF-β signaling and the role of CKB in TGF-β signaling in breast cancer cells.
